# A Light-Sheet-Based Imaaging Spectrometer to Characterize Acridine Orange Fluorescence within Leukocytes

**DOI:** 10.3390/diagnostics10121082

**Published:** 2020-12-12

**Authors:** Amy J. Powless, Sandra P. Prieto, Madison R. Gramling, Jingyi Chen, Timothy J. Muldoon

**Affiliations:** 1 Biomedical Engineering Department, University of Arkansas, Fayetteville, AR 72701, USA; apowless@uark.edu (A.J.P.); spprieto@uark.edu (S.P.P.); 2 Pat Walker Health Center, University of Arkansas, Fayetteville, AR 72701, USA; gramling@uark.edu; 3 Department of Chemistry and Biochemistry, University of Arkansas, Fayetteville, AR 72701, USA; chenj@uark.edu

**Keywords:** acridine orange, imaging spectrometer, fluorescence characteristics, leukocytes

## Abstract

Low-cost imaging systems that utilize exogenous fluorescent dyes, such as acridine orange (AO), have recently been developed for use as point-of-care (POC) blood analyzers. AO-based fluorescence imaging exploits variations in emission wavelength within different cell types to enumerate and classify leukocyte subpopulations from whole blood specimens. This approach to leukocyte classification relies on accurate and reproducible colorimetric features, which have previously been demonstrated to be highly dependent on the cell staining protocols (such as specific AO concentration, timing, and pH). We have developed a light-sheet-based fluorescence imaging spectrometer, featuring a spectral resolution of 9 nm, with an automated spectral extraction algorithm as an investigative tool to study the spectral features from AO-stained leukocytes. Whole blood specimens were collected from human subjects, stained with AO using POC methods, and leukocyte spectra were acquired on a cell-by-cell basis. The post-processing method involves three steps: image segmentation to isolate individual cells in each spectral image; image quality control to exclude cells with low emission intensity, out-of-focus cells, and cellular debris; and the extraction of spectra for each cell. An increase in AO concentration was determined to contribute to the red-shift in AO-fluorescence, while varied pH values did not cause a change in fluorescence. In relation to the spectra of AO-stained leukocytes, there were corresponding red-shift trends associated with dye accumulation within acidic vesicles and at increasing incubation periods. The system presented here could guide future development of POC systems reliant on AO fluorescence and colorimetric features to identify leukocyte subpopulations in whole blood specimens.

## 1. Introduction

A leukocyte differential count is a subset of a complete blood count (CBC), which is among the most frequently ordered blood tests in clinical laboratories [1,2,3]. Abnormalities in the leukocyte count have been used to diagnose a myriad of blood disorders and diseases including infections (bacterial, viral, and parasitic), inflammation and allergic reactions, and is also used to monitor drug response to various treatments such as chemotherapy [1,2,3,4]. For instance, an increase in lymphocytes is associated with viral infections, and an increase in neutrophils is associated with acute inflammation [1]. A leukocyte differential count is commonly reported as a proportion of leukocyte subpopulations, where a three-part leukocyte differential reports the proportion of lymphocytes, monocytes, and granulocytes [3]. Currently, there are two accepted methods to perform this test: a manual enumeration of a blood smear or by using an automated hematology analyzer. The manual enumeration approach involves the identifying and classifying 100 stained cells using a light microscope by a trained technician, which can be time and labor intensive, and can result in subjective operator error [5,6,7,8]. To overcome these limitations, automated hematology analyzers based on flow cytometry techniques (electrical impendence, forward and side light scattering and immunostaining) have become the “gold standard” due to their high-throughput (10,000–25,000 cells per sec), high specificity and sensitivity, reliability, and rapid diagnostics [5,9,10,11]. However, due to their large size, cost at upwards of $20,000, requirement of several proprietary reagents, and the requirement of trained technicians, these analyzers are unavailable in both developed and developing rural and remote areas and economically-challenged countries [2,3,7,9].

In recent years, point-of-care (POC) testing has aimed to increase test accessibility to a patient, reaching individuals in rural clinics, general practices, ambulatory care, and resource limited areas [5,12,13] POC blood analyzers have been designed to be portable (handheld and transportable tabletop systems) [2,14], cost less than $1 per-test [15], minimally invasive requiring less than 100 µL of blood, requiring little to no training to operate, and reduced turnaround time for the results [5,12,16]. A common design approach for POC blood analyzers is to utilize fluorescence-based imaging systems capable of imaging undiluted whole blood with minimal sample preparation within a microfluidic chip (single cell flow) [10] or on a slide to mimic a blood smear [16,17,18,19]. Although many POC systems have had success in detecting the three leukocyte subpopulations, the monocyte population remains a challenge to distinguish since they represent only 2–8% of the total leukocyte population [20]. For instance, a clinical hematology analyzer capable of counting over 10,000 cells per second compared to a POC miniaturized system capable of counting 100 cells total would result in 200–8000 and 2–8 monocytes available for detection, respectively. 

Fluorescent dyes are being used in POC systems to aid in distinguishing these leukocyte populations since many are biocompatible, long-term chemically stable, and can be tailored to target specific molecules [21]. The most distinct advantage of fluorescence readout is the high contrast provided by the emitted light against a blank background. Acridine orange (AO) is a fluorescent, rapid, vital dye with amphipathic properties capable of staining live, nucleated cells; therefore, the use of AO can minimize the number of reagents such as RBC lysis or other associated sample preparation steps [10,22]. Its fluorescence emission is dependent on its cellular content, which preferentially stains DNA via intercalation (λ_max_ = 525 nm), and red-shifts in emission when staining RNA via electrostatic interactions and when trapped in acidic vesicles (λ_max_ = 650 nm) [10,22,23]. When AO diffuses into acidic vesicles (approximately pH 5), such as lysosomes, it becomes protonated and trapped within the vesicles leading to dye accumulation and stacking of the AO molecules which result in a red-shifted emission wavelength [22,24,25,26,27]. Consequently, AO can be used to distinguish the three leukocyte populations due to their intracellular differences. Lymphocytes contain a large nucleus and little cytoplasm resulting in AO fluorescence emission predominately in the green wavelength region, granulocytes contain many acidic vesicles resulting in the largest red-shift amongst all cell populations, and AO emission from monocytes lies in between the green and red wavelength regions [22,28].

Variations in AO concentration and incubation time can cause an emission wavelength shift in whole blood samples, which may result in inaccurate cell counts [29]. Recent designs of POC blood analyzers using AO measure the absolute red and green fluorescence emission using either filter sets for green and red wavelength ranges [16,17,19], such as 525/50 nm and 650/50 nm [10], or color cameras which interpolate the color sensor elements into red, green, blue channels via a Bayer filter [29]. The detected mean red and green fluorescence intensities or the ratio of the two, called the red-to-green ratio (RG ratio), are then used to classify the cells. When the RG ratio is plotted as a histogram, three peaks appear: two distinct peaks representing lymphocytes and granulocytes, and a small peak in between representing monocytes [29,30]. However, we have previously demonstrated that variations in AO concentration and incubation time can cause an emission wavelength shift in whole blood samples, which resulted in variable cell counts using the RG ratio method [29]. Improved understanding of how the POC-motivated staining procedure can affect the colorimetric features of the leukocyte populations could help to guide the development of future POC systems. Furthermore, the variability in RG ratio measurements highlights the gap in knowledge of the behavior of AO in leukocytes based on these colorimetric features alone; therefore, the complete fluorescence emission spectra could elucidate differences between the leukocyte populations. 

The fluorescence emission spectra of an AO-labeled cell can be acquired using a spectrometer, which measures the spectrum of emitted light using a diffraction grating [31]. In contrast to single point detectors, imaging spectroscopy collects a two-dimensional image, where one dimension records the spatial distribution of fluorescence, and the second dimension records the intensity of emission across a range of wavelengths. The use of spectral imaging can be beneficial when quantifying fluorescence from individual leukocytes, as opposed to collecting the spectra of bulk fluid containing all leukocyte populations [32]. Imaging spectroscopy could be applied to understand and model the behavior of AO within leukocytes under a range of realistic POC staining conditions and protocols.

Here we present a light-sheet based imaging spectrometer fluorescence platform with an automated spectral extraction method as an investigative tool to extract spectral features from AO-stained leukocytes. This information will be used to further understand the colorimetric features implemented in POC devices to distinguish leukocyte subpopulations in a three-part leukocyte differential test. To replicate the conditions of live cell staining of whole blood using AO in POC devices, few pre-processing steps were applied (such as cell separation methods and RBC lysis) prior to collecting image data. Overall, we observed the location in the spectra, along with the shape of the spectral curve, where the red and green fluorescence in peaks used to calculate RG ratios occur. These results exhibit the specific details in the colorimetric features that contribute to the fluorescence emission in AO-stained leukocytes that are interpolated by the Bayer filter used in color cameras and in bandpass filters that target specific wavelengths. These results demonstrate the potential of imaging spectroscopy to elucidate the behavior of dyes within leukocytes and to guide future iterations of POC devices, such as determining the optimal optical and staining parameters or optimizing the bandpass filter selection to improve the separation between the three leukocyte populations. 

## 2. Materials and Methods

### 2.1. Light-Sheet Based Imaging Spectrometer Fluorescence Platform

**Figure 1:**
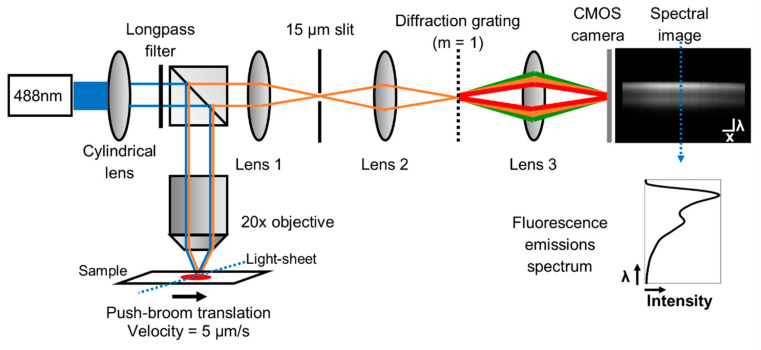
Schematic of the light-sheet based imaging spectrometer fluorescence platform using a push-broom translation method. From left to right, a 488 nm LED is emitted through a 200 mm cylindrical lens to generate a linear light-sheet. In an epifluorescence configuration, the light-sheet is transmitted through a 500 nm longpass filter into a 20× Nikon objective lens to excite the sample. The sample is translated under the light-sheet at a rate of 5 µm/s. The scale bars represent λ = 50 nm and x = 15 µm. The fluorescence emission spectrum is extracted from a vertical line of pixels across the image and can be plotted as wavelength versus fluorescence intensity. Image contrast enhanced for publication.

In order to quantify the spectrum of individual, AO-stained cells, we developed a light-sheet based imaging spectrometer fluorescence platform. A schematic of the light-sheet based imaging spectrometer is demonstrated in Figure 1. In an epitaxial illumination geometry, a 488 nm laser (FTEC2450-V20SFN, Blue Sky Research) is passed through a cylindrical lens (f = 200 mm, LJ1653RM-A, Thorlabs, Inc., Newton, NJ, USA) to form a light-sheet excitation beam that passes through the back aperture of a 20x Nikon Plan Fluor objective (NA = 0.50, Nikon Instruments, Inc.) onto the sample with an average power of 18.4 mW. Emitted light from the sample is collected by the objective, filtered through a 500 nm longpass filter (FEL0500, Thorlabs, Inc.), and imaged via a 100 mm achromatic doublet lens (AC254-100-A, Thorlabs, Inc.) onto a 15 µm slit aperture (S15R, Thorlabs, Inc.). The slit is then imaged via a 30 mm achromatic doublet lens (AC254-030-A, Thorlabs, Inc.) onto a transmission diffraction grating, (300 grooves/mm, 17.5° groove angle, GT25-03, Thorlabs, Inc.). The first order diffraction is then imaged via a 50 mm achromatic doublet lens (AC254-050-A, Thorlabs, Inc.) onto a monochrome 8-bit Flea 3 CMOS camera (FL3-U3-32S2M, FLIR Systems, Inc., Wilsonville, OR, USA). The fluorescence emission spectrum is therefore imaged as a function of linear position and can be extracted for further analysis on a cell-by-cell basis. Overall, the system has an optical magnification of 52.6 and an estimated spectral resolution of 8.9 nm. For the linear translation of the sample, a stepper motor and controller (TST101, Thorlabs, Inc.) were connected to a linear actuator (ZST225B, Thorlabs, Inc.) on a mechanical stage controlled by the APT motion-control software from Thorlabs. The controller was translated at a velocity of 5 µm/s covering 300 µm/min during image acquisition.

### 2.2. Linear Translation of the Sample

**Figure 2:**
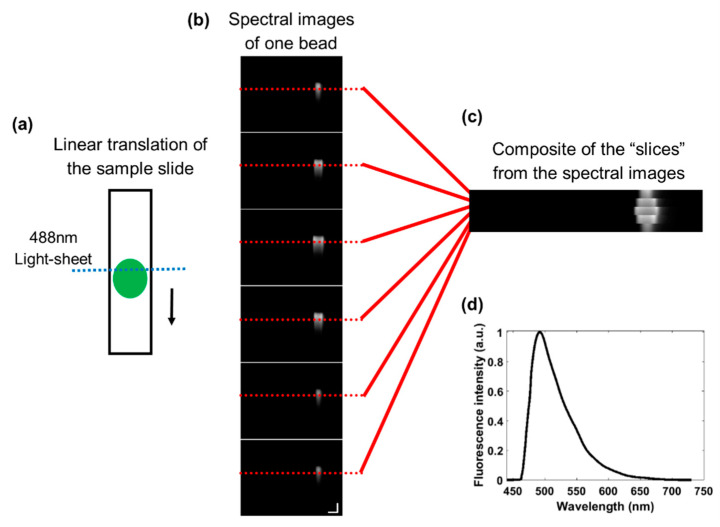
The linear translation process to acquire spectral images of whole objects. (**a**) A glass slide containing a sample is linearly translated under the 488 nm light-sheet illumination at a rate of 5 µm/s. (**b**) A series of spectral images from a single green fluorescence bead. The scale bar represents 15 µm (*x*-axis) and 50 nm (*y*-axis). (**c**) A composite image of the single green fluorescence bead by combining “slices” (red lines) from the spectral images. (**d**) The normalized spectrum extracted from the bead in (**c**). Image contrast enhanced for publication.

The process to acquire spectral images using the light-sheet based imaging spectrometer fluorescence platform involves the linear translation of a slide containing a monolayer of sample under the 488 nm light-sheet illumination on a mechanical stage controlled by a linear actuator (Thorlabs, Inc.) (Figure 2a). Green polystyrene fluorescence beads (15 μm, λ_em_ = 480 nm, F21010, Molecular Probes) were used to calculate the spatial resolution of the system and illustrate the linear translation and spectral images acquired. Spectral images were acquired by a CMOS camera with the *x*-axis representing the linear position across the slide in microns and *y*-axis representing the spectra produced by the diffraction grating. As the slide is translated, spectral images are acquired at multiple linear sections of the sample, resulting in multiple images associated with individual objects (Figure 2b). For visual examination, linear sections (“slices”) of the images can be compiled into a composite image (Figure 2c). The extracted spectra from the green bead is demonstrated in Figure 2d. A monolayer of AO-stained leukocytes was used for primary data acquisition.

### 2.3. System Validation and Calibration

**Figure 3:**
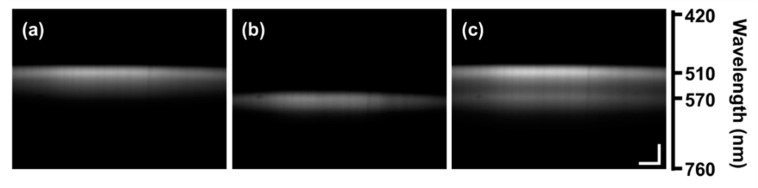
Spectral images of fluorescent dyes: (**a**) fluorescein, (**b**) rhodamine B, and (**c**) a mixture of fluorescein and rhodamin B at 1:10 volume ratio. The scale bars represent λ = 50 nm and x = 15 µm. Image contrast enhanced for publication.

The fluorescence emission spectra of known fluorescent dyes were acquired using the light-sheet based imaging spectrometer and a commercial spectrometer (USB2000 + UV-VIS, Ocean Optics, USA). The fluorescent dyes included fluorescein sodium salt (λ_em_ = 515 nm, F6377, Sigma-Aldrich, St. Louis, MO, USA) at a concentration of 1 mg/mL in distilled water, rhodamine B (λ_em_ = 583 nm, R6626, Sigma-Aldrich) at a concentration of 5.8 × 10^−4^ g/mL in distilled water, and a 1:10 by volume mixture of both. Representative spectral images of each dye solution are shown in Figure 3.

**Figure 4:**
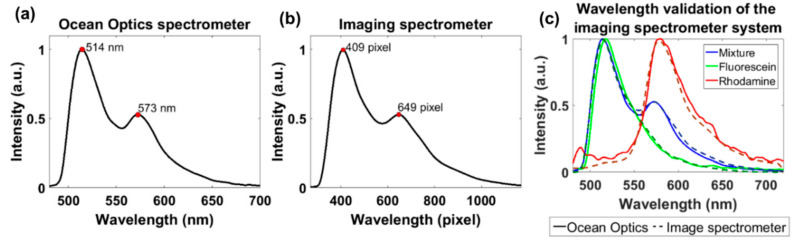
Mapping of the pixel location in the spectral images to wavelength. Normalized spectra acquired by the (**a**) Ocean Optics spectrometer and (**b**) light-sheet based imaging spectrometer of the fluorescence dye mixture: fluorescein and rhodamine B. The first and second peaks in each plot correspond to fluorescein and rhodamine B with a peak emission of 514 nm and 573 nm, respectively. These peaks relate to the pixel location at 409 and 649 in the spectral images acquired by the light-sheet based imaging spectrometer. (**c**) The comparison between both systems was used to validate the assignment of the wavelengths (nm) to each pixel location in the spectral images.

In the spectra of the dye mixture, the two fluorescence emission peaks corresponding to each dye were used to correlate and convert the pixel location in the spectral images to wavelength in nanometers (Figure 4a,b). The emission wavelength for each peak was acquired by the commercial system (514 nm and 573 nm); therefore, the pixel locations for the peak in the spectra acquired by the image spectrometer were assigned their corresponding nanometer value. Once this range was determined, the values in between those points were interpolated using evenly spaced increments (Figure 4). This pixel location-to-wavelength conversion and an alignment method (see next Section) were repeated daily to calibrate the system and monitor for system instability. To validate the system, using the calibrated wavelength scale and alignment, the spectra from all the dye solutions were compared to the spectra acquired by the commercial spectrometer (Figure 4c).

### 2.4. Alignment of the Illumination Pattern in each Spectral Image

**Figure 5:**
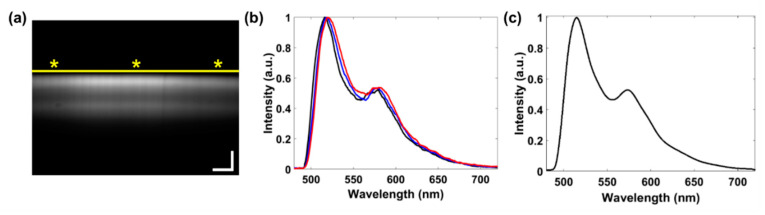
Alignment method to compensate for optical aberrations in the light-sheet-based imaging spectrometer. (**a**) A spectral image of a fluorescence dye mixture of fluorescein and rhodamine B. The scale bar represents 15 µm (*x*-axis) and 50 nm (*y*-axis). The yellow line illustrates the variations in the illumination pattern. (**b**) Individual normalized spectral curves for three vertical pixel lines corresponding to the locations in the spectral image denoted by asterisks in (**a**). (**c**) The aligned, averaged, and normalized spectral curve from the spectral image. (Image contrast enhanced for publication.).

An alignment method was developed to compensate for the optical aberrations in the in-house built system that could cause undesirable variance in the spectra. An example spectral image demonstrating the effects of the optical aberration is shown in the Figure 5a by the yellow line above the illumination pattern, where the signal is lower on the edges of the image than the center making an arched pattern. To perform the alignment, the first peak on each spectral curve in the image acquired from each vertical line of pixels was located. In Figure 5b, three example spectral curves are used to demonstrate the 4 nm misalignment associated with corresponding areas on the spectral image (asterisks in Figure 5a). Each peak was aligned to the leftmost peak by calculating the difference between the two and subtracting that value by the peak location in each curve. The final spectra in Figure 5c was the average between all aligned spectra in the Figure 5b. This method was used to align the spectra in all images associated with each cell.

### 2.5. Characterization of Acridine Orange Fluorescence

Fluorescence emission spectra of varied AO staining conditions (pH and concentration) were acquired using the light-sheet based imaging spectrometer to characterize the red-shift in fluorescence that occurs within AO-stained cells. Six solutions of 100 µg/mL acridine orange hemi (zinc chloride) salt (A6014, Sigma-Aldrich) (AO) were prepared at different pH conditions (pH = 3.0, 3.8, 5.0, 6.0, 7.3, and 8.0). Since the pH of 3.8 and 7.3 have been used in studies involving AO-stained cells [33,34], the range of pH values used in this study were chosen to represent a range surrounding those biologically relevant values. Five solutions of AO at pH of 5.0, the pH when AO becomes protonated within vesicles, [25] were prepared with concentrations of 100 µg/mL, 250 µg/mL, 500 µg/mL, 750 µg/mL, and 1000 µg/mL. Spectral images were acquired using a 2000 ms exposure and gain of 20 dB. Due to the high gain causing noise, a moving average filter was applied to the extracted spectra, and then the spectra was normalized for direct comparison between each AO staining condition. 

### 2.6. Blood Collection and Staining of Whole Blood

Under the approval of the Institutional Review Board at the University of Arkansas (IRB #13-06-759) and following informed consent, whole blood was collected by a trained phlebotomist from 5 healthy volunteers over the age of 18. A maximum of 4 mL of whole blood was collected via venipuncture in a K2 EDTA 7.2 mg vacutainer collection tube (367862, Becton, Dickinson and Company, Franklin Lakes, NJ, USA). Eight µL of whole blood was mixed with 8 µL of 20 µg/mL acridine orange hemi(zinc chloride) salt (pH = 7.34, A6014, Sigma-Aldrich) and incubated for 3, 5, and 7 min following the results reported by Powless, et al. Spectral images were acquired in 2 min intervals per incubation time, referred to as incubation periods henceforth [29]. Image acquisition settings included a 3 sec integration time and 22 dB gain, where the system was capable of counting an average of 5 cells per min with a maximum signal-to-background ratio (SBR) of 2.5. The method to calculate the SBR is described in the following section. 

### 2.7. Automated Spectral Extraction Algorithm

**Figure 6:**
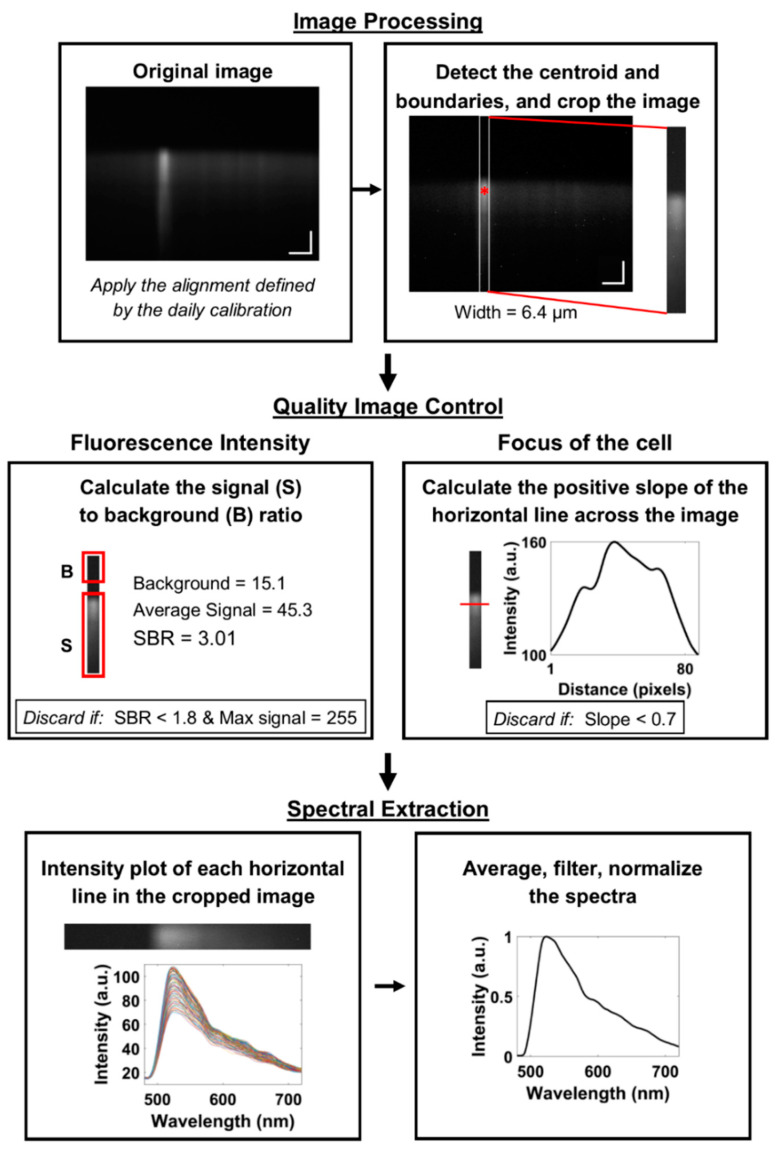
Flow diagram of the automated spectral extraction method. This consisted of three main steps: (**top**) image processing to detect objects in the image, (**middle**) quality image control to account for optical variability in the focus and illumination, and (**bottom**) spectral extraction to yield the complete spectra for each object. Image contrast enhanced for publication.

A detailed flow diagram of the automated spectral extraction algorithm, designed to detect cells within an image and extract the spectra, is presented in Figure 6. This involves three steps performed in MATLAB (MathWorks, Natick, MA, USA): image processing, quality image control, and spectral extraction.

### 2.8. Image Processing

The spectral images were processed to isolate the cells in each image and discard cellular debris. Initially, the spectral images were binarized to perform image segmentation techniques in MATLAB, such as “imclose” to fill in gaps within the cells and “bwareaopen” to exclude small cells associated with cellular debris. The location of the centroid and edge locations, called boundaries, of the cells were detected using the MATLAB function “regionprops”, which found the center of mass and edges in the binary image of the region that returned a value of 1. Additionally, the distance between the centroids were used to discriminate between cells in the same field of view. Any overlapping objects defined as being larger than 170 pixels wide, or objects with pixels touching the edge of the image, were excluded. The images were cropped to the boundary locations to isolate only the spectra associated with each cell. The top and bottom edges of the cropped image were fixed at the full length of the image to include the whole spectral range.

### 2.9. Quality Image Control

**Figure 7:**
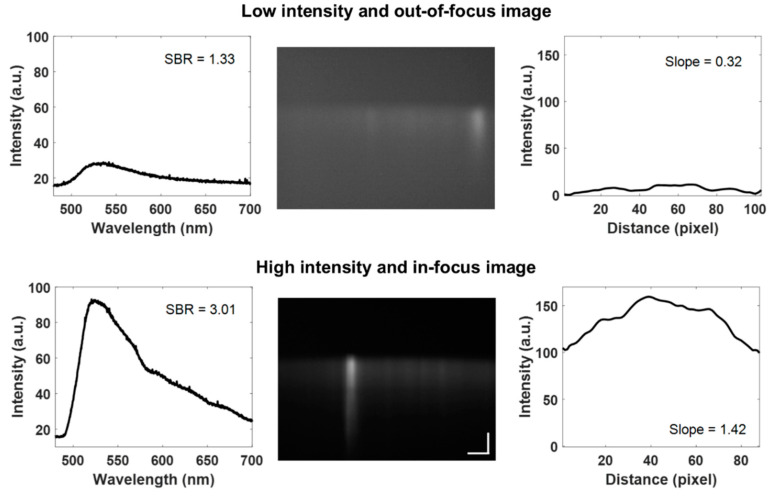
Quality control example images and corresponding parameters such as low and high-intensity images and out-of-focus and in-focus images. (Image contrast enhanced for publication.).

Quality control was incorporated into the automated extraction algorithm to reject cellular debris, low signal-to-background spectra, and out-of-focus cells (Figure 7). Using the edge locations, the width of each cell was calculated. Leukocytes range from 6 to 14 µm in size, although some monocytes can exceed this range [20]. Using this generalized range and images with known groups of cells, any cell with widths greater than 13 µm were considered groups of cells or cellular debris and were excluded. Images with a maximum fluorescence intensity greater than 254 were defined as having oversaturated pixels, since the available range in these 8-bit images is 0–255, and thus excluded. Additionally, the signal-to-background ratio (SBR) was used to exclude low fluorescence intensity images. The upper region of the cropped image with no signal present was averaged and considered the background. The lower region starting just above the start of the fluorescence signal in the center of the image was averaged and considered the main signal. These regions were fixed for every image. Images with SBR less than 1.8 were defined as low intensity, which contributes to significant spectral noise, therefore they were excluded. Next, the slope was calculated along one edge of the cell in the spatial direction (*x*-axis) at a fixed position in all images to evaluate the focus of the image assuming cells in focus will have an abrupt gradient in the fluorescence signal between the background and edge. In-focus images were considered to have a slope greater than 0.7 based on the average for all images (Figure 7).

### 2.10. Spectral Extraction

The spectra were extracted from the images that met the acceptance criteria defined in the previous sections. For visualization, in Figure 6 under Spectral Extraction section the cropped image was rotated horizontally for a direct comparison to the extracted spectral curves in the plot below the image. The spectral curves were generated by plotting the pixel location along horizontal lines of the image against the fluorescence intensity at each pixel. Although there is some spatial variation in the spectra acquired from within each cell—due to the various green to red emission wavelengths generated by AO accumulation in intracellular compartments—we wished to analyze the overall average emission spectrum for each cell. Due to poor fluorescence signal causing noise and variations in the spectra, a moving average filter was applied, and the filtered spectra was normalized.

## 3. Results

**Figure 8:**
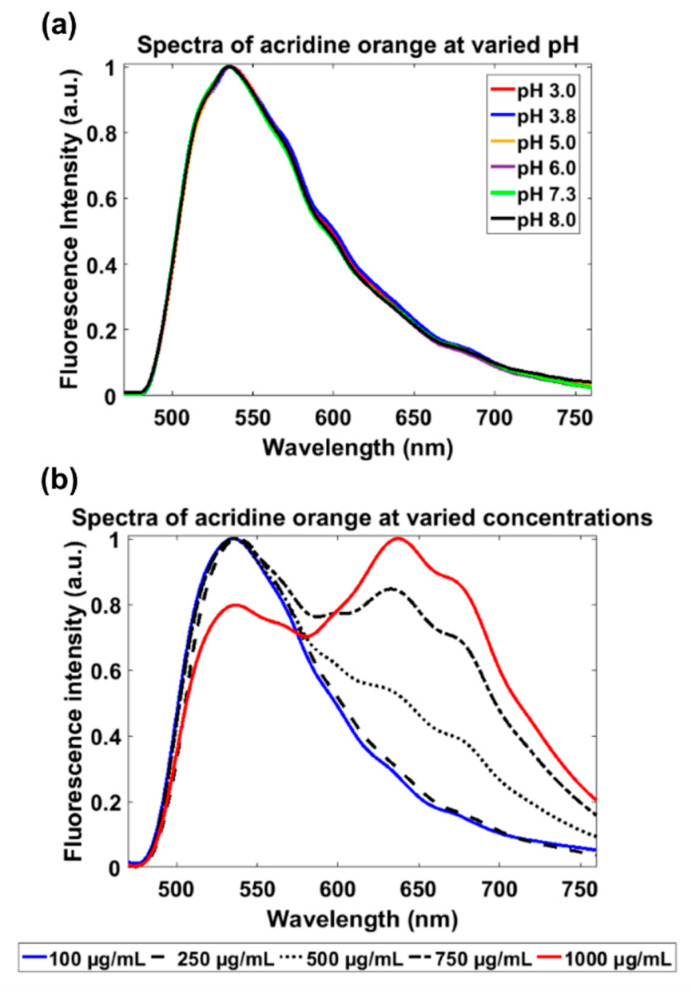
Spectra extracted from spectral images of AO solutions at varied pH values and concentrations. (**a**) A plot of the emission spectra of six AO solutions at pH values of 3.0, 3.8, 5.0, 6.0, 7.3, and 8.0. (**b**) A plot of the emission spectra of five AO solutions, with a fixed pH of 5, at the concentrations of 100 µg/mL, 250 µg/mL, 500 µg/mL, 750 µg/mL, and 1000 µg/mL.

The extracted spectra acquired by the light-sheet based imaging spectrometer of AO solutions at different staining conditions are presented in Figure 8. In Figure 8a, there was a 1nm peak shift from 535 nm to 536 nm between the lowest and highest pH values. In Figure 8b, a minor 1nm peak shift occurred from 535 nm to 536 nm, however there was a substantial increase in fluorescence intensity from 0.54 to 1.0 in red fluorescence around 630 nm between the lowest and highest AO concentrations. 

**Figure 9:**
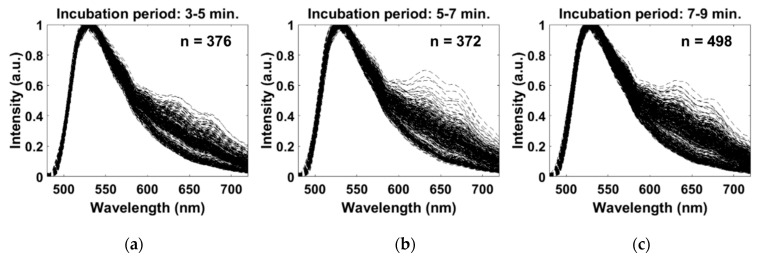
Spectra extracted from spectral images of AO-stained leukocytes for three staining incubation periods: (**a**) 3–5 min, (**b**) 5–7 min, and (**c**) 7–9 min.

The extracted spectra acquired by the light-sheet based imaging spectrometer of AO-stained leukocytes is presented in Figure 9 for the incubation periods of (a) 3–5 min, (b) 5–7 min, and (c) 7–9 min. Qualitatively, there are two distinct regions of the spectra. For all incubation periods, the first region of the spectra has one distinct peak at 529 nm, which is within 4 nm of the emission maximum of AO-stained DNA. The second region, surrounding 650 nm, does not have a distinct singular peak; although, it contains the greatest difference between each spectrum. In comparison between the three incubation periods, there is an observable increase in the number of spectra with greater fluorescence intensity in the second region over time, which indicates an increase in red fluorescence. However, between the last two incubation periods, there is no observable increase in the number of spectra with greater red fluorescence intensity.

## 4. Discussion

AO fluorescence is known to red-shift due to stacking of AO monomers via dye accumulation [24,25,27]. This predominately occurs within acidic vesicles in the cells where there is a decrease in pH causing the protonation of AO resulting in the entrapment and accumulation of AO [25,27]. To demonstrate this phenomenon and validate that the red-shift is not a result of the pH difference, six AO solutions at varied pH conditions and five AO solutions with varied concentrations were investigated. No peak shift of the AO fluorescence spectra was observed with increased pH (Figure 8a), while the increase of AO concentration could cause substantial red-shift of the AO fluorescence peak (Figure 8b). As the concentration increased to 500 µg/mL, a peak at approximately 630 nm began to appear, indicating a red-shift in fluorescence likely due to the accumulation and aggregation of AO molecules. 

In this study, we have established two regions in the emission spectra of AO-stained leukocytes that correspond to the green and red fluorescence emission often used in POC devices. Commonly, these devices implement filter sets to isolate key center wavelengths of the spectra that correspond to the maximum emission wavelengths of AO bound to varied cellular components (525 nm, 650 nm) [10,16,17]. Alternatively, color cameras are being implemented in POC devices to simultaneously acquire three color channels (red, green, and blue) via a Bayer filter without additional optical components or detectors. Both design approaches aim to simplify the fluorescence signal into two specific colorimetric features to allow for automated classification, however the whole spectra could elucidate the underlying behavior of AO fluorescence within each subpopulation of leukocytes. We and others have demonstrated previously that AO staining of intact, whole blood specimens, following by fluorescence colorimetric imaging using a Bayer filter-equipped low-cost microscope, is highly sensitive to the specific details of the staining protocol and imaging procedures used [17,29]. In particular, the timing of when the staining and imaging is performed has a profound impact on the measured red-to-green fluorescence emission properties of these cells. The light-sheet based imaging spectrometer approach we have described here enables high-resolution study of AO-induced emission spectra, which can guide further development of whole blood AO staining and imaging protocols in future POC devices. 

Using our light-sheet based imaging fluorescence platform with an automated spectral extraction algorithm as an investigative tool, we have extracted the spectra associated with the colorimetric features used in fluorescence-based POC systems that apply AO as a contrast agent, as demonstrated in Figure 9. Overall, the spectra acquired during all incubation periods exhibited two regions of the spectra. The first region, with a peak centered at 529 nm for all cells, correspond to the maximum emission wavelength of AO when bound to DNA (525 nm), which is prevalent in the nuclei. Since all leukocytes are nucleated, this region of the spectra did not contribute to the differentiation of the cells, however it illustrates the ability of the spectra to account for the AO fluorescence in the nuclei regardless of the potential masking effect caused by the fluorescence in the cytoplasm above the focal plane. The second region of the spectra (centered on 650 nm), demonstrated the most variability between the cells, which could be associated with the difference in the cytoplasmic content, such as RNA and acidic vesicles [26]. Although the variations in the second region did not yield three distinct groups, there are observable trends that could correspond to lymphocytes and granulocytes. The group of spectra with the lowest fluorescence intensity in the second region, are likely to correspond to the lymphocyte population, which are known to emit predominately green fluorescence when stained with AO due to their DNA-rich nucleus and minimal cytoplasm [27,28]. The group of spectra with the greatest increase in intensity in the second region, are likely to correspond to the granulocyte population due the red-shift in fluorescence of AO when accumulated within acidic vesicles [25].

For the monocyte population, the spectra was expected to be in the middle of the other groups, described previously, since they are known to have similar morphological and colorimetric features to the other populations stained with AO. However, as a whole, the spectra for the presumed monocyte population were indistinguishable from the other populations. This could be related to the limited total cell count, which monocytes exhibit population fractions of 2% to 8% in normal samples [20]. This challenge was seen in the system described here, which yielded a cell count of approximately 350 to 500 per incubation interval tested and 7–40 monocytes available to detect. In order to address this concern and improve the number of cells counted per unit time in our light-sheet based imaging spectrometer device, certain modifications to this architecture could be performed, such as increasing detector sensitivity by using an electron multiplying charge-coupled (EMCCD) camera and using higher laser power to deliver more signal to the detector. 

In summary, we have developed a light-sheet based imaging spectrometer with an automated spectral extraction algorithm to quantify, on a cell-by-cell basis, the specific spectral changes seen in leukocytes when stained with AO under conditions which simulate POC testing. The results presented here could be used to guide future development of POC systems using AO, specifically by focusing on the spectral window around 650 nm which accounts for the majority of the differences among the three major leukocyte subpopulations. This approach also may suggest the potential of the light-sheet based imaging spectrometer architecture could be used to investigate individual intracellular changes within AO-stained leukocytes or the behavior of other dyes.

## 5. Conclusions

AO is known to emit unique colorimetric features for three subpopulations of leukocytes (lymphocytes, monocytes, and granulocytes), which are dependent on the cellular content of each population. This study represents the proof-of-concept of a research-specific technique to elucidate differences between the entire spectra of AO-stained leukocytes, rather than mean red and green fluorescence or RG ratio commonly used in POC blood analyzers. The results from the combination of our light-sheet based imaging spectrometer fluorescence platform and an automated spectral extraction algorithm demonstrate the potential of this system to be used as an investigative tool which could guide future POC devices or be built upon to explore individual intracellular changes within AO-stained leukocytes.
